# More Insight into BDNF against Neurodegeneration: Anti-Apoptosis, Anti-Oxidation, and Suppression of Autophagy

**DOI:** 10.3390/ijms18030545

**Published:** 2017-03-03

**Authors:** Shang-Der Chen, Chia-Lin Wu, Wei-Chao Hwang, Ding-I Yang

**Affiliations:** 1 Department of Neurology, Kaohsiung Chang Gung Memorial Hospital, Kaohsiung 83301, Taiwan; chensd@adm.cgmh.org.tw; 2 Institute for Translation Research in Biomedicine, Kaohsiung Chang Gung Memorial Hospital, Kaohsiung 83301, Taiwan; 3 College of Medicine, Chang Gung University, Taoyuan 33302, Taiwan; 4 Program in Molecular Medicine, National Yang-Ming University and Academia Sinica, Taipei 11221, Taiwan; ta730112@hotmail.com; 5 Institute of Brain Science and Brain Research Center, National Yang-Ming University, Taipei 11221, Taiwan; 6 Department of Neurology, Taipei City Hospital, Taipei 11221, Taiwan; huangshiaowei@gmail.com

**Keywords:** 3-nitropropionic acid, p62, reactive oxygen species, sestrin2, sulfiredoxin

## Abstract

In addition to its well-established neurotrophic action, brain-derived neurotrophic factor (BDNF) also possesses other neuroprotective effects including anti-apoptosis, anti-oxidation, and suppression of autophagy. We have shown before that BDNF triggers multiple mechanisms to confer neuronal resistance against 3-nitropropionic acid (3-NP)-induced mitochondrial dysfunction in primary rat cortical cultures. The beneficial effects of BDNF involve the induction of anti-oxidative thioredoxin with the resultant expression of anti-apoptotic B-cell lymphoma 2 (Bcl-2) as well as erythropoietin (EPO)-dependent stimulation of sonic hedgehog (SHH). We further revealed that BDNF may bring the expression of sulfiredoxin, an ATP-dependent antioxidant enzyme, to offset mitochondrial inhibition in cortical neurons. Recently, we provided insights into another novel anti-oxidative mechanism of BDNF, which involves the augmentation of sestrin2 expression to endow neuronal resistance against oxidative stress induced by 3-NP; BDNF induction of sestrin2 entails the activation of a pathway involving nitric oxide (NO), cyclic guanosine monophosphate (cGMP)-dependent protein kinase (PKG), and nuclear factor-κB (NF-κB). Apart from anti-apoptosis and anti-oxidation, we demonstrated in our most recent study that BDNF may activate the mammalian target of rapamycin (mTOR) with resultant activation of transcription factor c-Jun, thereby stimulating the expression of p62/sequestosome-1 to suppress heightened autophagy as a result of 3-NP exposure. Together, our results provide in-depth insight into multi-faceted protective mechanisms of BDNF against mitochondrial dysfunction commonly associated with the pathogenesis of many chronic neurodegenerative disorders. Delineation of the protective signaling pathways elicited by BDNF would endow a rationale to develop novel therapeutic regimens to halt or prevent the progression of neurodegeneration.

## 1. Introduction

Neurotrophic factors are critical ligands for neuronal cells to proliferate, differentiate, and grow during developmental stages and play important roles to maintain survival, network connectivity, and neuronal plasticity in adult brains. Among the neurotrophic factors expressed in the brain, the neurotrophin family, which includes neurotrophin 3 (NT-3), neurotrophin-4 (NT-4), nerve growth factor (NGF), and brain-derived neurotrophic factor (BDNF), have been extensively investigated. BDNF and its well-known transmembrane receptor, tropomyosin-related kinase B (TrkB), have gained much attention in neuropsychiatric disorders [1,2]. BDNF has multipotent effects on neural development and exerts synaptic plasticity that prompts circuit formation and cognitive function.

Increasing evidence suggests that synaptic dysfunction is a crucial pathophysiological feature of neurodegenerative disorders [3]. Synaptic dysfunction and synapse loss in human brain tissues are evident in such neurodegenerative disorders as Alzheimer’s disease (AD) [4], Parkinson’s disease (PD) [5], and Huntington’s disease (HD) [6]. The concept that BDNF-based synaptic repair may carry therapeutic potential in neurodegenerative diseases [3] is beyond the scope of the present discussion and detailed reviews regarding synaptic dysfunction in neurodegenerative diseases can be seen in several previous publications [7,8,9].

It is well appreciated that low extent of reactive oxygen species (ROS) and reactive nitrogen species (RNS) are critical for maintenance of neuronal function [10]. On the other hand, however, excessive ROS/RNS is proposed to take part in a detrimental role in the pathogenesis of neurodegenerative disorders. Mounting evidence indicates that neurodegenerative diseases are characterized by increased levels of oxidation markers, including lipid, protein, and DNA, or lower levels of antioxidant capability in the brain [11]. Heightened oxidative stress is involved in the pathophysiology of neurodegenerative diseases such as AD, PD, and HD [12]. A diverse cascade of events including calcium overload, excitotoxicity, mitochondrial dysfunction, and apoptotic processes contribute to oxidative stress-mediated neurodegeneration [10]. In addition to small-molecule antioxidants, evidence indicates that neurotrophic factors such as BDNF may also avert neuronal damage caused by oxidative stress [10].

Alteration of the activities and levels of BDNF have been shown in several neurodegenerative disorders. Among them, with gain- and loss-of-function experiments, a possible mechanistic link between BDNF with HD has been established [13]. BDNF levels are reduced in HD; studies in HD animal models and human tissues show reduced cortical BDNF mRNA and protein levels as compared to the control groups [2]. The extent of BDNF trafficking is decreased in the presence of the HD mutation in vitro [14]. Studies using animal models of the aforementioned human diseases to increase BDNF levels in the brains will ultimately influence the clinical treatment of these disorders. “BDNF therapies” such as augmenting BDNF levels by gene or protein delivery, enhancing BDNF signaling through various methods, and developing small molecules to target BDNF production are proposed [2].

3-Nitropropionic acid (3-NP) is a well-known toxin of Indigofera that irreversibly inhibits succinate dehydrogenase activity in the complex II of the mitochondrial electron transport chain [15]. 3-NP may cause neurodegeneration through oxidative stress, energy dysfunction, and excitotoxicity; all are pertinent to HD pathogenesis. Indeed, 3-NP is used to investigate the molecular mechanisms of cell death caused by mitochondrial dysfunction and neurodegeneration for HD. Systemic administration of 3-NP in rats results in a gradual progress and excitotoxic cell death in striatal neurons, which is associated with dystonia and other abnormal motor behaviors [16]. Similar 3-NP-induced pathological phenotypes were also observed in nonhuman primates [17,18]. In this review, we briefly introduce the recent advance in the molecular mechanisms of the BDNF-mediated protective effects against neuronal dysfunction induced by 3-NP, including our previous studies [19]. We also discuss our recent works on sestrin2 [20], which is an acute stress response protein, as well as autophagy inhibition [21] in the paradigm of BDNF and 3-NP in fetal cortical neurons. Clarification of BDNF-mediated protective actions against 3-NP-induced neurotoxicity may aid in developing a therapeutic regimen for HD in which mitochondrial dysfunction plays a critical role in the pathogenesis of this devastating disease [20].

## 2. Molecular Mechanisms for Brain-Derived Neurotrophic Factor (BDNF)-Induced Protective Effects against 3-Nitropropionic Acid (3-NP) Toxicity

BDNF has been recognized as an important growth factor that exerts a beneficial effect on neuronal function under various stressful conditions [22]. BDNF binding triggers TrkB dimerization and autophosphorylation of tyrosine residues in the receptor, which in turn recruits adaptor proteins and transduction molecules that activate three main downstream phosphorylation cascades including phospholipase Cγ (PLCγ), phosphatidylinositol 3-kinase (PI3-K)/Akt, and mitogen-activated protein kinases (MAPKs) pathways that together act as the predominant regulators by BDNF/TrkB [22]. Downstream of these regulators, several targets may be activated to contribute to the neuroprotective effects of BDNF. These include transcription factors such as actin-binding proteins, cyclic adenosine monophosphate (cAMP) response-element binding protein (CREB), and transcriptional regulators such as the mammalian target of rapamycin (mTOR). BDNF exerts neuroprotective effects by antagonizing *N*-methyl-d-aspartate receptor (NMDAR) triggered excitotoxicity, promoting dendritic regeneration, and possesses anti-apoptotic effects via B-cell lymphoma 2 (Bcl-2) protein and/or by post-translational modifications of apoptosis-related proteins such as Bad and Bim [19]. Impaired BDNF signaling may contribute to a wide range of neurologic and psychiatric disorders, including stroke, epilepsy, trauma, as well as neurodegenerative disorders such as AD, PD, and HD [2,22].

Apart from its well-known neurotrophic actions and anti-apoptotic effects, anti-oxidative effects of BDNF may also contribute considerably to its protective characteristics in various experimental paradigms mimicking neurodegenerative conditions, which have gained less attention. BDNF increases the expression levels of superoxide dismutases (SODs) and glutathione reductase in cultured hippocampal neurons of rats [23]. BDNF may exert its protective effects by regulating superoxide anion homeostasis during an experimental model of temporal lobe status epilepticus [24]. Moreover, BDNF also reduces the level of tyrosine nitration, a marker for oxidative protein damage [25]. It was shown that BDNF could upregulate mitochondrial uncoupling protein 2 (UCP2) and restore the reduced mitochondrial electron coupling capacity in rostral ventrolateral medulla of spontaneously hypertensive rats [26]. Because 3-NP is a mitochondrial toxin known to trigger oxidative stress, BDNF-mediated protective effects against 3-NP-induced toxicity in neurons are speculated. It has been demonstrated that BDNF protects cortical neurons from 3-NP-induced apoptotic cascades triggered by mitochondrial dysfunction, activation of caspase-3, and chromatin condensation by decreasing both total and mitochondrial Bim levels [27]. BDNF can rescue 3-NP-induced reduction of CREB phosphorylation and CREB-binding protein levels in cortical neurons [28]. Despite these reports, however, the detailed molecular mechanisms of BDNF-mediated neuroprotection against 3-NP toxicity remain to be fully clarified.

In the past decade, we have been working in this field in an attempt to reveal the neuroprotective mechanisms of BDNF [19,20,21,29,30,31,32], as well as other trophic factors such as oncostatin M that is beyond the range of the current review [33], against neurodegeneration using a pharmacological model with 3-NP challenge to mimic oxidative stress. We showed that BDNF-dependent protective effects against 3-NP toxicity could be eliminated by a nonselective nitric oxide (NO) synthase (NOS) inhibitor l-nitroarginine methylester (l-NAME). These effects may occur partially through the activation of the signaling pathways involving NOS/NO, thioredoxin, cyclic guanosine monophosphate (cGMP)-dependent protein kinase (PKG), and Bcl-2 [31]. We further demonstrated that BDNF-mediated 3-NP resistance in cortical neurons also counts on the effects of sonic hedgehog (SHH), a mammalian member of the hedgehog family which regulates the polarity of the central nervous system as well as proliferation, self-renewal, and survival of neuronal cells [29]. BDNF enhanced SHH expression at both mRNA and protein levels and, moreover, the expression of SHH mRNA heralded the increase of its protein products following exposure to BDNF, suggesting participation of a transcriptional mechanism; more significantly, BDNF-dependent protection was eradicated by cyclopamine, a SHH pathway inhibitor, indicating a causal relationship between SHH induction and BDNF neuroprotection [29].

Erythropoietin (EPO) is a 34-kDa cytokine and is vital for the survival and differentiation of red blood cells during erythropoiesis [34]. It was noticed that cells in the central nervous system, including neurons, glial cells, and cerebral endothelial cells, also produce EPO and express EPO receptor [35]. EPO possesses a neuroprotective effect against assorted insults in the nervous system [36]. Because carbamylated EPO may activate SHH [37], we determined whether BDNF-dependent SHH expression and 3-NP resistance demand prior induction of EPO. We showed that BDNF activated EPO expression at both mRNA and protein levels [30]. BDNF-mediated 3-NP resistance and SHH induction were both eliminated by the soluble EPO receptor (sEPO-R), which functions as an EPO inhibitor capable of competing with the endogenous EPO-R for its ligand EPO. Recombinant rat EPO can induce SHH expression and lessen 3-NP neurotoxicity. This study demonstrated that SHH expression and 3-NP resistance under BDNF treatment entail prior induction of EPO. These results thus determine a signaling cascade of “BDNF → EPO → SHH → 3-NP resistance” in rat cortical neurons [30].

It was reported that BDNF could augment the antioxidant enzymes activities such as glutathione peroxidase, glutathione reductase, and superoxide dismutases (SODs) in cultured cortical neurons [23] and serves widespread roles in regulating energy homeostasis [38]. However, the underlying protective mechanisms of BDNF in relation to its antioxidant activities still await full delineation. The relationship between c-Jun and BDNF in various neurological insults was reported before [39,40]. However, whether BDNF can induce c-Jun activation and the probable biological functions is not well understood. During investigation of the BDNF effects on activation of c-Jun N-terminal kinases (JNKs) and their downstream target c-Jun, we found that BDNF substantially enhances the expression of c-Jun without inducing JNK phosphorylation [32]. c-Jun is a well-known component of the activator protein 1 (AP-1) transcription factor complex. Sulfiredoxin, a reductase that has recently been identified as an AP-1 target gene [41], can reduce hyperoxidized peroxiredoxin in an ATP-dependent manner into the catalytically-active thiol form [42]. Sulfiredoxin, under an excessive oxidative condition, catalyzes the development of a sulfinic acid phosphoric ester on peroxiredoxins that can be reduced by thiol equivalents such as the NADPH-thioredoxin reductase system. Therefore, we established the notion that c-Jun-dependent sulfiredoxin expression conveys the protective effects of BDNF against neurotoxicity induced by 3-NP in rat primary cortical cultures [32].

## 3. Sestrin2 Induction Conducts the Antioxidant Effects of BDNF against Mitochondrial Inhibition

Sestrin2 was first recognized as one of the hypoxia-inducible genes, hence named hypoxia-inducible gene 95 (*Hi95*) that may be elicited by prolonged hypoxia [43]. Sestrin2 is crucial for the maintenance of metabolic homeostasis [44]. Previously, the induction of sestrin2 was suggested to offer cytoprotective capability against diverse stressors, such as ischemia and hydrogen peroxide, via the restoration of overoxidized functional peroxiredoxins under excessive oxidative stress [43]. Recently, the potential antioxidant ability of sestrin2 in the nervous systems is emerging. Thus, a recent report showed the defensive activity of sestrin2 against neurotoxicity induced by 1-methyl-4-phenylpyridinium (MPP^+^), a mitochondrial complex I inhibitor, in neuroblastoma SH-SY5Y cells [45]. We have demonstrated before that sestrin2 induced by amyloid β-peptide (Aβ), a neurotoxic protein component of senile plaques observed in the brains of AD patients, exerts its functions as an endogenous protective mediator against Aβ-induced neurotoxicity through the regulation of autophagy [46]. Similarly, transient global brain ischemia can induce sestrin2 expression and confer a neuroprotective effect against ischemic injury in the hippocampal CA1 subfield [47]. Sestrin2 also regulates oxidative stress-related neuropathic pain in peripheral nerve damage [48]. All these findings indicate the protective effects of sestrin2, at least partly, through lessening oxidative stress and/or the promotion of autophagy in the experimental models of neurodegenerative diseases.

Regardless of the recognized mediators downstream of BDNF from our previous studies [29,30,31,32] such as thioredoxin, Bcl-2, EPO, SHH, and sulfiredoxin to protect cortical neurons against 3-NP-induced toxicity, the versatile defensive mechanisms of BDNF, particularly in those of antioxidant action, remain to be further revealed. The potential connection between sestrin2 and BDNF has never been established. In our recent study [20], we validated the hypothesis that BDNF may increase sestrin2 expression to convey the protective effect against oxidative stress induced by 3-NP and demonstrated the molecular mechanisms of BDNF to induce sestrin2 expression in rat primary cortical cultures. In this work, we demonstrated that the NO/PKG-1 signaling pathway is engaged in BDNF-mediated sestrin2 induction [20]. It was known that PKG is a member of AGC family of serine/threonine protein kinases. The AGC group is termed due to the name of protein kinase A, G, and C families (PKA, PKG, PKC) in the role of cytoplasmic serine/threonine kinases that are governed by secondary messengers such as cAMP (PKA) or lipids (PKC) [49]. All PKG family members are stimulated by augmenting the cellular cGMP level via the action of guanylyl cyclases, a well-known process triggered through NO signaling [50]. PKG signaling is vital for many biological functions such as contraction of the intestinal smooth muscle, bone growth, cardiac contractility, axon guidance, and erectile dysfunction [51].

Although we have demonstrated that the BDNF/NO/cGMP/PKG signaling pathway mediates sestrin2 induction in rat primary cortical neurons, the transcription factor accountable for those BDNF effects awaits to be identified. Several possible transcription factors known to induce sestrin2 expression in a variety of model systems such as p53 [52], hypoxia-inducible factor-1 (HIF-1) [53], c-Jun [54], and nuclear factor (erythroid-derived 2)-like 2 (Nrf2) [55] were tested in our study; however, the results revealed that sestrin2 activation by BDNF was not mediated by these transcription factors. Using the transcription factor searching software TFsearch, we found promising binding sites for nuclear factor-κB (NF-κB) in the promoter region of sestrin2 gene. To confirm whether NF-κB mediates BDNF-dependent sestrin2 induction, several experimental approaches including cellular fractionation followed by Western blotting, double immunofluorescence confocal microscopy, siRNA-mediated gene knockdown, and chromatin immunoprecipitation (ChIP) assay were conducted. We noted that BDNF increases NF-κB subunits p65 and p50 in nuclear protein fractions and also boosts their physical association to form the heterodimeric NF-κB complex for BDNF-stimulated sestrin2 expression in cortical neurons. BDNF-induced NF-κB activation requires NO/sGMP/PKG activity and, interestingly, the PKG-1 indeed forms a protein complex with p65/p50 and together translocates into the nucleus to trans-activate sestrin2 expression in the cortical neurons exposed to BDNF. Thus, we provided evidence to support a novel signaling pathway of “BDNF/TrkB → NO/cGMP/PKG → NF-κB → sestrin2” that ultimately play a role in suppressing the generation of ROS and neurotoxicity from 3-NP exposure in cortical neurons [20].

## 4. Roles of p62 in Mediating BDNF-Dependent Neuroprotection against Mitochondrial Inhibition via Autophagy Suppression

Autophagy, meaning “self-devouring”, is induced in response to nutrient deprivation for the degradation of macromolecules through the lysosomal system to advocate cell survival by either eradicating the cell of damaged organelles and toxic pathogens or by restoring metabolites for energy supply for growth support under nutrient-limited conditions [56]. The mTOR is a central regulator of autophagy [57,58,59]. Under nutrient-rich conditions, mTOR is activated to trigger protein synthesis while suppressing autophagy; in stressful growth conditions, mTOR is inactivated to inhibit global protein synthesis while triggering autophagy. Another type of autophagic degradation, namely the selective autophagy, involves ubiquitin-binding proteins such as p62/sequestosome-1 (p62/SQSTM1; hereby referred to as p62) [60]. Several lines of evidence have shown that, in selective autophagy, p62 plays a role in the development of large aggregates/inclusions known as p62 bodies or sequestosomes [61], which enlist and carry ubiquitinated cargo to the autophagosome for degradation through connection of p62 with microtubule-associated protein 1A/1B-light chain 3 (LC3)-II, the membrane components of the autophagosomes [62,63]. 3-NP is known to induce autophagy both in vivo and in vitro. In the in vivo paradigms, biochemical analyses of the rat striatum treated with 3-NP via stereotaxic injection show activation of autophagy based on increased levels of beclin 1 and LC3-II [64]. In the in vitro paradigms, 3-NP has been shown to induce autophagy in SH-SY5Y cells [65]. However, whether BDNF-mediated neuroprotective effects against 3-NP involve the suppression of autophagy and, if so, the potential underlying mechanisms remain to be further delineated. In our most recent report, we examined the hypothesis that BDNF may suppress 3-NP-induced autophagy to exert its neuroprotective effects by inducing the expression of p62 in primary cortical neurons [21]. Using the LC3-II/LC3-I ratio as an index for autophagy and double immunofluorescence confocal microscopy to co-localize LC3-positive signal with mitochondria and lysosomes for respectively mitophagy and autolysosome formation, we found that 3-NP enhances autophagy in primary cortical neurons, which is attenuated by BDNF preconditioning; moreover, BDNF time-dependently induced the expression of p62 [21]. We have shown BDNF-induced c-Jun in cortical neurons [32]. In this work, we found that c-Jun knockdown in part attenuated BDNF-mediated p62 induction; upstream of p62, BDNF triggered phosphorylation of mTOR and its downstream mediator p70 ribosomal protein S6 kinase (p70S6K) [21]. In addition to suppressing p62 induction, the mTOR inhibitor rapamycin also partially suppressed BDNF-induced c-Jun expression. Together, our results revealed that BDNF inhibits 3-NP-induced autophagy via, at least in part, the mTOR/c-Jun-dependent induction of p62 expression, together contributing to neuroprotection against mitochondrial inhibition.

## 5. Experimental Evidence Supporting BDNF-Mediated Neuroprotection against 3-NP Toxicity in Animal Models

Because all our mechanistic studies on the neuroprotective mechanisms associated with BDNF preconditioning were conducted in primary cortical neurons in vitro, whether these results can be duplicated in the animal models in vivo remains to be confirmed. At present, there are no reports on the neuroprotective effects of exogenously applied BDNF in the 3-NP-treated animals. Nevertheless, it has been shown that treatments with phosphodiesterase-5 inhibitors (sildenafil or vardenafil), which selectively blocks degradation of cGMP, improved neurologic scores and reduced loss of striatal lesion volumes that were produced by the systemic infusion of 3-NP in rats and, importantly, the striatal expression of BDNF was significantly increased in the sildenafil-treated rats [66]. This in vivo study appears to be consistent with our contention that the cGMP/PKG pathway is important for the neuroprotective effects against 3-NP toxicity in vitro [20,31]. Previous studies have also demonstrated that 3-NP exposure may reduce the tissue contents of BDNF in vivo. For example, chronic administration of 3-NP reduced BDNF levels in the striatum, but not in the cortex, in the lesioned mouse brains [67]. Furthermore, several studies also reported up-regulation of BDNF levels by other neuroprotectants in the 3-NP-treated animals. Thus, 3-NP-induced deficits in motor behaviors can be reversed by Praeruptorin C, an effective component in the root of *Peucedanum praeruptorum* Dunn that is a traditional Chinese medicine; more importantly, Praeruptorin C up-regulated several proteins, including BDNF, in the striatum of the 3-NP-treated mice [68]. Memantine, a NMDA blocker well known for its protective effect against various neurodegenerative diseases, improved 3-NP-induced motor deficits and concomitantly increased the expression of BDNF in the brains of 3-NP-treated mice [69]. Cannabigerol, a nonpsychotropic phytocannabinoid, improved motor deficits and preserved striatal neurons against 3-NP toxicity accompanied by improvement in the levels of antioxidant defenses that were reduced by 3-NP; interestingly, cannabiogerol also enhanced the expression of BDNF in the R6/2 mice mimicking the pathology of HD [70]. In addition to small-molecule compounds, stem cells may also confer neuronal resistance against 3-NP via the up-regulation of BDNF. Transplantation of human neural stem cells (hNSCs) alleviated 3-NP-mediated striatal damages and improved motor performance; further analysis revealed the expression of BDNF in vitro in the cultured hNSCs as well as the expression and secretion of BDNF in vivo from the grafted hNSCs [71]. Transplantation of mesenchymal stem cells (MSCs) improved motor impairments and reduced the enlargement of the lateral ventricles, both induced by 3-NP, along with increased striatal labeling of BDNF in the brains of MSC-transplanted rats [72]. Overall, these previous studies appear to support the contention that, in vivo, BDNF also plays a critical role in attenuating 3-NP toxicity. Whether the protective mechanisms of BDNF are similar to those observed in vitro, as has been proposed by us using primary cortical neurons, requires further investigation.

## 6. Conclusions and Future Prospect

**Figure 1:**
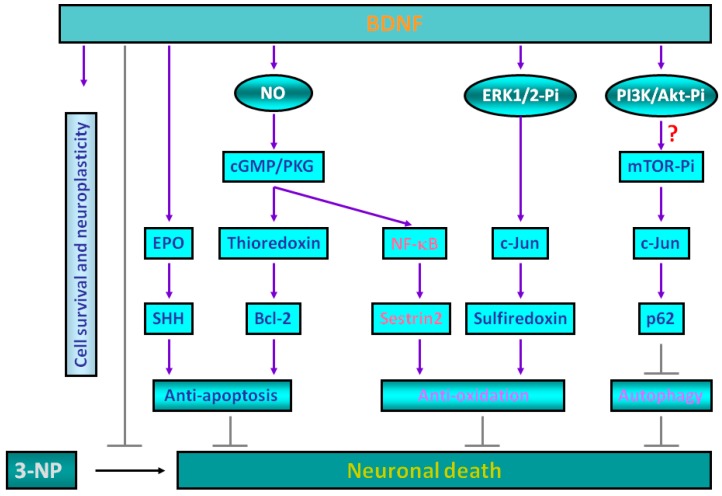
Multiple neuroprotective mechanisms including anti-apoptosis, anti-oxidation, and autophagy suppression are inducible by brain-derived neurotrophic factor (BDNF). The neuroprotective actions of BDNF against mitochondrial dysfunction associated with exposure to 3-nitropropionic acid (3-NP) in cortical neurons involve a number of different molecular mediators. First, BDNF may induce sonic hedgehog (SHH) via erythropoietin (EPO) that together contribute to its anti-apoptotic actions [29,30]. Second, BDNF preconditioning also triggers the induction of nitric oxide (NO) with the enhanced production of cGMP as well as increased expression of PKG-1, together leading to the expression of anti-oxidative thioredoxin and anti-apoptotic Bcl-2 proteins [19,31]. Third, the BDNF-induced NO/cGMP/PKG pathway also contributes to the activation of the redox-sensitive transcription factor NF-κB; indeed, upon BDNF exposure, PKG-1 forms a protein complex with the NF-κB subunits p65 and p50 and translocates into the neuronal nucleus to drive the expression of sestrin2, which attenuates cellular ROS contents produced by 3-NP [20]. Fourth, BDNF stimulates the phosphorylation of extracellular signal-regulated kinase-1/2 (ERK1/2) with subsequent induction of another transcription factor c-Jun, thereby leading to the expression of an ATP-dependent antioxidant enzyme sulfiredoxin [32]. Finally, BDNF also activates mTOR and induces c-Jun to drive the expression of p62 that suppresses the 3-NP-induced activation of autophagy and neuronal death [21].

Multiple and perhaps reciprocally regulated signaling cascades exist to mediate BDNF-dependent neuroprotective effects that together convey neuronal protection against 3-NP-induced mitochondrial dysfunction and the resultant neurotoxicity (Figure 1). These pathways include anti-apoptosis, anti-oxidation, as well as emerging new mechanisms such as regulation of autophagy. Demarcation of the protective signaling cascades induced by BDNF and the translation of the results that stemmed from basic studies into a clinically curative intervention for HD and other neurodegenerative disorders will be the next challenging step.

## Abbreviations

3-NP	3-nitropropionic acid
Aβ	amyloid β-peptide
AD	Alzheimer’s disease
AGC family	protein kinases A, G, and C (PKA, PKG, PKC)
AP-1	activator protein 1
Bcl-2	B-cell lymphoma 2
BDNF	brain-derived neurotrophic factor
cAMP	cyclic adenosine monophosphate
cGMP	cyclic guanosine monophosphate
ChIP	chromatin immunoprecipitation
CREB	cAMP response-element binding protein
EPO	erythropoietin
ERK1/2	extracellular signal-regulated kinase-1/2
HD	Huntington’s disease
HIF-1	hypoxia-inducible factor-1
hNSCs	human neural stem cells
JNKs	c-Jun N-terminal kinases
l-NAME	l-nitroarginine methylester
MAPKs	mitogen-activated protein kinases
MPP^+^	1-methyl-4-phenylpyridinium
MSCs	mesenchymal stem cells
mTOR	mammalian target of rapamycin
NF-κB	nuclear factor-κB
NGF	nerve growth factor
NMDAR	*N*-methyl-d-aspartate receptor
NO	nitric oxide
NOS	nitric oxide synthase
Nrf2	nuclear factor (erythroid-derived 2)-like 2
NT3	neurotrophin 3
NT-4	neurotrophin 4
p70S6K	p70 ribosomal protein S6 kinase
PD	Parkinson’s disease
PI3-K	phosphatidylinositol 3-kinase
PKA	cAMP-dependent protein kinase
PKG	cGMP-dependent protein kinase
PLCγ	phospholipase Cγ
RNS	reactive nitrogen species
ROS	reactive oxygen species
SHH	sonic hedgehog
SODs	superoxide dismutases
TrkB	tropomyosin-related kinase B
UCP2	uncoupling protein 2
